# *In Vitro* Hepatic Trans-Differentiation of Human Mesenchymal Stem Cells Using Sera from Congestive/Ischemic Liver during Cardiac Failure

**DOI:** 10.1371/journal.pone.0092397

**Published:** 2014-03-18

**Authors:** Dillip Kumar Bishi, Santosh Mathapati, Kotturathu Mammen Cherian, Soma Guhathakurta, Rama Shanker Verma

**Affiliations:** 1 Stem Cells and Molecular Biology Laboratory, Department of Biotechnology, Indian Institute of Technology Madras, Chennai, India; 2 Stem Cells and Tissue Engineering Laboratory, International Centre for Cardiothoracic and Vascular Diseases, Frontier Lifeline, Chennai, India; 3 Department of Engineering Design, Indian Institute of Technology Madras, Chennai, India; University of Udine, Italy

## Abstract

Cellular therapy for end-stage liver failures using human mesenchymal stem cells (hMSCs)-derived hepatocytes is a potential alternative to liver transplantation. Hepatic trans-differentiation of hMSCs is routinely accomplished by induction with commercially available recombinant growth factors, which is of limited clinical applications. In the present study, we have evaluated the potential of sera from cardiac-failure-associated congestive/ischemic liver patients for hepatic trans-differentiation of hMSCs. Results from such experiments were confirmed through morphological changes and expression of hepatocyte-specific markers at molecular and cellular level. Furthermore, the process of mesenchymal-to-epithelial transition during hepatic trans-differentiation of hMSCs was confirmed by elevated expression of E-Cadherin and down-regulation of Snail. The functionality of hMSCs-derived hepatocytes was validated by various liver function tests such as albumin synthesis, urea release, glycogen accumulation and presence of a drug inducible cytochrome P450 system. Based on these findings, we conclude that sera from congestive/ischemic liver during cardiac failure support a liver specific microenvironment for effective hepatic trans-differentiation of hMSCs *in vitro*.

## Introduction

Liver disease is one of the leading causes of mortality worldwide, accounting for two million deaths every year [1]. Liver, an organ with highest inbuilt regenerative capacity, cannot restore its damaged tissues in end-stage liver diseases. Although most common causes of hepatic dysfunctions leading to liver failure are excessive alcohol usage, toxic injury and viral infection, there are incidences of hepatic abnormality observed in cardiac failure [2]. Major causes of such complications are chronic congestion due to heart failure and ischemic hepatitis from acute circulatory impairment. The prevalence of liver dysfunction in cardiac failure is approximately 15–65% [3]. There are cases of cardiac-failure-associated liver congestion/ischemia leading to hyperbilirubinemia, sometime referred as “cardiac jaundice” [4], [5].

Present status of liver salvation in end-stage liver diseases is orthotopic liver transplantation, but it has serious limitations like donor availability and organ rejection [6]. Cellular therapy using hepatocytes are promising, but inadequate cell source for hepatocyte transplantation is a major hurdle [7]. Therefore, regenerative therapy using stem cells looks promising as it can provide a clinically feasible alternative to liver transplantation due to the unique plasticity, clonality and differentiation potential of stem cells towards functional hepatocytes *in vitro* and *in vivo* [8], [9]. The major categories of stem cells that have been implicated for hepatic regeneration are resident hepatic stem cells (oval cells, hepatocytes, foetal liver cells, etc.) and extra-hepatic stem cells [such as embryonic stem (ES) cells, induced pluripotent stem cells (iPSCs), adult stem cells from bone marrow, cord blood and adipose tissue)] [10]. Resident hepatic stem cells (oval cells) are the best candidates to repopulate damaged livers [11], but they are scarce and are unfavourable for large-scale expansion during clinical application [12]. ES cells possess tremendous hepatogenic potential, but ethical issues and risk of tumour formation restricts their therapeutic application [8]. Similarly, iPSCs though hold great promises for hepatocyte generation, safe and effective iPSCs for use in cell therapy are yet to be developed [13]. Adult bone marrow stem cells are reported as a potential cell source for liver regeneration [14] and can generate hepatocytes *in vitro* and *in vivo* [15], [16]. Clinical trials involving autologous bone marrow mesenchymal stem cell (MSC) therapy for liver diseases showed substantial improvements in liver functionality [17]–[19]. Since hMSCs could be harvested from the patient’s own marrow and can effectively give rise to functional hepatocytes, it has tremendous potential for end-stage liver failure treatment.

MSCs have been trans-differentiated effectively towards hepatocyte-like cells *in vitro* using an array of commercially available recombinant growth factors [hepatocyte growth factor (HGF), epidermal growth factor (EGF), fibroblast growth factor (FGF)], cytokines [Oncostatin M (OSM)] and chemical compounds (nicotinamide, dexamethasone, insulin etc.) by inducing either as a cocktail [20] or in a sequential manner [21], [22]. In fact, HGF alone is found to be sufficient for hepatic differentiation of MSCs [23]. However, hepatic inductions with such recombinant growth factors are not optimal for clinical applications due to their bacterial origin and in most cases they are not free of endotoxins. Thus a natural source of hepatogenic factors, readily available from patients, would be ideal as a conditioned culture system to augment hepatic differentiation of stem cells with suitable clinical relevance. There have been well known reports of usage of liver failure sera and cholestatic sera upon hepatogenic induction of bone marrow stem cells [24]–[28], which describe the potential role of hepatogenic factors (including HGF) released from hepatocytes during liver damage or cholestasis. Serum levels of HGF increase in patients with a variety of liver diseases [29], [30] as well as in cardiovascular diseases such as acute myocardial infarction, hypertension and congestive heart failure [31]–[34]. In the present study, our primary goal was to evaluate the effectiveness of a novel hepatogenic conditioned sera collected from patients with cardiac-failure-associated secondary hyperbilirubinemia (jaundice) on hepatic trans-differentiation potential of human bone marrow MSCs. The patient sera used for hepatic induction were assessed for the presence of hepatogenic factors (such as HGF) and we could achieve functional hepatocytes with such novel hepatogenic conditioned culture system.

## Materials and Methods

### Assessment of Clinical and Biochemical Profiles of Patients with Cardiac-failure -associated Congestive/ischemic Liver

#### Study Approval

This study was reviewed and approved by the Institutional Ethics Committee of International Centre for Cardiothoracic and Vascular Diseases, Frontier Lifeline hospital, Chennai, India.

#### Patient and Control Cohorts

Twenty seven patients with cardiac-failure-associated congestive/ischemic liver with symptoms of secondary jaundice (hyperbilirubinemia) were recruited for this study from the critical care unit of International centre for cardiothoracic and vascular disease, Frontier Lifeline hospital, Chennai. In addition a control group, consisting of 27 volunteers who were age, gender and ethnically matched to the patient group was recruited. The investigation conforms to the principles outlined in the Declaration of Helsinki [35]. Written informed consents were obtained from all participants before inclusion in the study and the initiation of any study related procedures. The inclusion criteria of the patient group were: presence of chronic cardiac complications leading to heart failure and have developed jaundice (total bilirubin ≥ 3.0). The inclusion criteria for the control group were: absence of a known coronary, valvular, or myocardial disease. Co-morbidities for coronary artery disease such as diabetes mellitus, hypertension, hyperlipidaemia, and smoking did not preclude recruitment. Exclusion criteria for all participants were: pregnancy, dialysis, and known or treated malignancies, viral infection, or drug induced liver dysfunction, hepatobiliary diseases, cirrhosis or alcoholic hepatitis. Patients were excluded if they had pre-existing liver disease or injury to the liver during trauma, any preexisting chronic medical condition (including hepatitis, organ system failure). Sera from both patients as well as control groups were collected and screened for microbial infections, hepatitis and endotoxin and stored in –80°C for further experiments.

### Clinical and Biochemical Profile of Patients with Cardiac-failure-associated Liver Dysfunction

All the clinically relevant data such as patient demographics, history of cardiac disease, etiology and the main precipitating cause of cardiac-failure-associated hyperbilirubinemia, cardiovascular risk factors as well as results of X-ray, echocardiographic and laboratory tests were collected from medical records. The patients were categorized into various cardiac disease groups such as ischemic heart disease (IHD), valvular heart disease (VHD), dilated cardiomyopathy (DCM) and congenital heart disease (CHD) based upon their signs and symptoms. Baseline laboratory data, particularly serum bilirubin (total and direct), albumin, alkaline phosphatase, alanine aminotransferase (ALT), aspartate aminotransferase (AST), lactate dehydrogenase (LDH), γ-glutamyl transpeptidase (GGT), alkaline phosphatase (ALP), urea, creatinine and haemoglobin tests were collected from medical record for each individuals on admission to hospital or during follow up. Based on liver function tests for serum enzymes (ALT, AST, GGT and ALP) and bilirubin level, the patients were categorized to have either hepatocellular or cholestatic profile as per earlier reports [36]. The concentration of HGF present in patient sera and normal sera was measured by ELISA (Human HGF Quantikine ELISA kit, R&D systems MN, USA) as per the manufacturer’s protocol.

### Hepatic Trans-differentiation of Human MSCs

#### Growth Characteristics of hMSCs in Presence of Cardiac-failure-associated Jaundiced Sera (in vitro Cytotoxicity Assay)

Three different sets of human MSC cell line were evaluated in this study, one acquired from Lonza (Allendale, NJ, USA) and two others isolated and expanded from patient bone marrow in our laboratory. Cell proliferation assay of hMSCs grown in various hepatogenic culture conditions reveals the overall impact of sera components (particularly bilirubin) on cell growth. Therefore, we evaluated the *in vitro* cytotoxic effect of different concentrations of patient sera (10% vs. 5% in DMEM) upon proliferation potential of MSCs. As a control, the impact of normal volunteer donor’s sera (10% and 5% in DMEM) on hMSC proliferation was evaluated. Cells grown in 10% FBS was used as positive control. Cell death assay was performed by annexin V-FITC/PI apoptosis detection kit (Cell Signaling Technology) as per manufacturer’s protocol. Cell proliferation assay was done by direct cell counting using trypan blue on every 3^rd^ day until 9 days and also by MTT based *in vitro* toxicology assay kit (Sigma) as per manufacturer’s protocol. An increase or decrease in cell number results in a concomitant change in the amount of formazan formed as measured spectrophotometrically, indicating the degree of cytotoxicity caused by the serum components.

#### Hepatic Trans-differentiation - Protocol Optimization

MSCs of passage 3 were expanded on collagen type-I coated 35-mm diameter 6-well plates (Nunc, Wiesbaden-Biebrich, Germany) till 80% confluence in Isocove’s modified Dulbecco’s medium (IMDM) containing 10% FBS and antibiotics. Hepatic transdifferentiation was carried out as per the standard protocol [21] with minor modifications. Briefly, the expanded hMSCs (1.5×10^4^ cells/cm^2^) were subjected to endoderm specification with 20 ng/ml EGF and 10 ng/ml FGF-4 for 48 hrs in serum free IMDM. Hepatic induction was carried out by treating hMSCs with either of the following group of hepatogenic culture systems/control group:

Group I: Pooled sera from patients with cardiac-failure-associated congestive/ischemic liver leading to jaundice (5% in IMDM)

Group II: Pooled sera from normal volunteers (5% in IMDM)

Group III: Commercially available well defined growth factor cocktail of HGF (20 ng/ml) + FGF-4 (10 ng/ml) + Nicotinamide (0.61 gm/L) (in serum free IMDM, positive control group)

Group IV: FBS (10% in IMDM, negative control group of uninduced hMSCs)

This step was continued for a period of 7 days with fresh induction media replenished twice weekly. The differentiating cells were then treated with maturation medium, consisting of IMDM supplemented with 20 ng/mL oncostatin M, 1 μmol/L dexamethasone, and 50 mg/mL ITS^+^ premix (Insulin, Transferrin, selenium, linoleic acid) till day 28.

#### Characterization of hMSC-derived Hepatocytes

*Morphological changes of trans-differentiated cells:* During hepatic trans-differentiation of hMSCs changes in morphology of differentiating cells in various induction groups were observed at various time points by phase contrast microscopy (Olympus, CX-41).

#### Immunofluorescence Analysis of Hepatic Markers Expression

The expression of hepatocyte specific markers (ALB, CY-18 and AFP) was analyzed for both induced and uninduced cells after 28 days of induction. HepG2 cells were used as positive control and uninduced hMSCs as negative control. The hMSCs–derived hepatocytes fixed in 100% methanol for 10 minutes at –20°C, washed and incubated in 3% BSA for 1hr at RT. The cells were then incubated with primary antibodies raised in mice against human albumin (1∶100) or cytokeratin-18 (1∶50) or α–fetoprotein (1∶200) overnight at 4°C. To confirm mesenchymal to epithelial transition happening during hepatic trans-differentiation of hMSCs, they were stained with primary antibody raised against human E-Cadherin raised in mice (a gift from Prof. Jonathan M. G. Higgins, Harvard Medical School, USA). Next day, cells were washed and incubated with secondary anti-mouse Alexafluor 488 (for albumin and cytokeratin-18) (1∶200) or Alexafluor 594 (for α-fetoprotein and E-Cadherin) (1∶200) for 1 hr in dark and counterstained with nuclear stain (Hoechst 33342) (1∶250). The stained cells were observed under a fluorescent microscope (Nikon TiE) and images were captured.

#### RNA Isolation and Real time-qPCR (RT-qPCR) Analysis for Hepatic Genes Expression

Total RNA was extracted from hMSCs-derived hepatocytes under various culture conditions, HepG2 cells and untreated hMSCs using TRI Reagent (Sigma) as per manufacturer’s protocol and quality and quantity of RNA was measured spectrophotometrically. For cDNA synthesis, 5 μg of total RNA was transcribed with MMLV-reverse transcriptase (Sigma). Quantitative real time-PCR for early hepatic genes such as AFP, HNF-4α and late hepatic genes such as CY-18 and ALB were carried out with Mastercycler ep *realplex* (Eppendorf, Germany) using SYBR Green JumpStart TaqReadyMix (Sigma). Assessment of mesenchymal-to-epithelial transition during hepatic differentiation of hMSCs was done by evaluating changes in expression of mesenchymal marker (SNAIL) and epithelial marker (CDH1, E-Cadherin). The primer sequence details are specified in Table S2. The experiment was performed in a 20 μl reaction mixture of 2× SYBR Green PCR Master Mix, 30 ng template cDNA and 5 pmol forward and reverse primers, for a total of 40 cycles with annealing temperature of 60°C. Relative quantification of gene expression in unknown samples was performed using the comparative C_T_ (2^–ΔΔCT^) method by normalizing with a housekeeping gene (glyceraldehyde-3-phosphate dehydrogenase) and untreated hMSCs were used as the calibrator. All reactions were performed in triplicate for statistical validation.

#### Flow Cytometry Analysis of hMSCs-derived Hepatocytes

To evaluate the efficiency of hepatic trans-differentiation, the total number of ALB^+^ cells in the hMSCs-derived hepatocytes and non-differentiating cell populations were quantified by flow cytometry. HepG2 cells were used as positive control and untreated hMSCs as negative control. Briefly, the cells after 28 days of induction were trypsinized and fixed with 4% para-formaldehyde (PFA) solution. Approximately, 10^6^ cells were aliquoted into FACS tube and permeabilized with 0.3% Triton-X, blocked with 3% BSA for 1 hr and incubated with primary monoclonal antibody against human albumin raised in mouse (Sigma) (1∶100) for 1hr at RT. The cells were washed and incubated further with FITC-conjugated anti-mouse secondary antibody (Sigma) (1∶400) for 1hr in dark. Similar procedure was adopted for quantifying endoglin^+^ (CD105) cells in the hMSCs-derived hepatocytes to crosscheck the status of undifferentiated cells. The stained cells were quantified by a flow cytometer (BDFACS Calibur) with 10000 events recorded for each sample and compared with the isotype control (human IgG/IgM).

#### Functional Characterization of Trans-differentiated Hepatocytes

Liver function tests were carried out to validate the functionality of hMSCs-derived hepatocytes. The amount of albumin secreted in the culture media collected at various time points (7^th^ day, 14^th^ day, 21^st^ day and 28^th^ day) was determined using human albumin ELISA quantitation set (Bethyl Laboratory, Montgomery, TX, USA) according to the manufacturer’s instructions. Since patient sera and control sera used in the conditioned media already contains albumin, its concentration was normalized and only the secreted albumin concentration was taken into account. The presence of insoluble stored glycogen content in the trans-differentiated cells was assessed by per-iodic acid Schiff’s (PAS) staining, for which the cells at day 28 were fixed with 4% PFA for 10 minutes. It was then oxidized with 1% per-iodic acid for 5 min, washed and incubated with Schiff’s reagent for 15 min. The cells were washed and counter stained with Mayer’s hematoxylin and observed under light microscope. Uninduced hMSCs were used as negative control. Urea concentrations in the hMSCs derived hepatocytes in different culture conditions and in controls were measured spectrophotometrically (Urea assay kit, Sigma) as per manufacturer’s instruction. To assess the presence of an inducible cytochrome P450 (CYP) system in the trans-differentiated hepatocytes, cells were induced with phenobarbital (1 mM) for 3 days and cytochrome P450-dependent pentoxyresorufin o-dealkylase activity (PROD) was performed. PROD assay without phenobarbital induction was used as experimental control. Fluorescence detection of PROD metabolism in the trans-differentiated hepatocytes and control cells were observed under microscope, which determined the CYP activity. Phenobarbital induced PROD activity (resorufin fluorescence) was quantified spectrophotometrically at excitation and emission wavelengths of 530 and 590 nm respectively [37].

### Statistical Analysis

The clinical data were presented as mean ± SD along with inter quartile range (IQR) or percentage (%), whichever is applicable. Continuous clinical variables were compared between the two groups by a two-sided unpaired t-test, whereas laboratory findings as well as HGF level was compared using Mann-Whitney U test, as the data were not normally distributed. The correlation between HGF level and the total bilirubin level was measured using the Spearman rank coefficient as the relationship was not linear. All the experiments were done in triplicate for statistical validation and reproducibility was assessed by repeating each experiment at least three times. Statistical differences among various groups were analysed using one- or two-way analysis of variance (ANOVA) followed by Tukey’s post hoc test with statistical package for social sciences (IBM SPSS statistics, version 19, NY, USA). Differences were considered statistically significant at *p*≤0.05.

## Results

### Clinical and Biochemical Profiles of Patients

#### Clinical Parameters

The baseline clinical characteristics and demographics of the patient and control groups are shown in Table 1. Among the patient group, 40.7% of subjects were presented with a history of ischemic heart disease (IHD) that includes most cases of coronary artery disease (double vessel disease and triple vessel disease) and ischemic cardiomyopathy. Around 66.6% of patients were having valvular heart diseases (VHD), represented mostly by rheumatic heart disease, severe mitral stenosis or mitral regurgitations. Remaining patients were either affected by dilated cardiomyopathy (DCM) (18.5%) or congenital heart diseases (CHD) (29.6%). A progressive deterioration of the cardiac function (chest pain, dyspnoea, ankle edema) was noted shortly before admission in 17 patients. Chest X-ray revealed that 5 patients had symptoms of cardiomegaly and three patients had levocardia and boot-shaped heart. Half of the patients were presented with severe CHF (chronic heart failure) (NYHA class III–IV, 51.85%) out of which five patients (18.5%) died of cardiac-failure-driven multi-organ failure with septic shock during hospitalization. The potential risk factors involved Type II diabetes mellitus, hypertension and hyperlipidaemia. Congestive hepatomegaly with ascites was observed in 7 patients, who were having no history of liver disease. Both the patient sera and control sera were complete microbe free and endotoxin free. Significant differences were observed in the laboratory findings (both haematological and biochemical parameters for liver function test) as well as clinical parameters (BP, heart rate and co-morbidities) between the patients and the control group.

**Table 1:** Baseline characteristics of patients and control group*^a^*.

Parameters	Cardiac-failure-associated hyperbilirubinemia patient group		Control group (without cardiovascular disease)		*P*-value
	Mean ± S.D. (or %)	IQR	Mean ± S.D. (or %)	IQR	(Mann-Whitney U test)
**Gender** (male)	19 (70.3%)		22 (81%)		0.349
**Age** (years)	42.2±13.2	14–63	44.4±12.2	19–68	0.531
**Underlying heart disease**			NA		NA
Ischemic heart disease (%)	8 (29.6%)				
Valvular heart disease (%)	11 (40.7%)				
Dilated cardiomyopathy (%)	3 (11.1%)				
Congenital heart disease (%)	5 (18.5%)				
NYHA class I (%)	3 (11.1%)				
NYHA class II (%)	10 (37%)				
NYHA class III/IV (%)	14 (51.85%)				
**Symptoms and signs**					
Heart rate (bpm)	79±12	66–104	72±6.3	65–82	0.01
Systolic blood pressure (mm Hg)	129.3±18.3	100–170	119.1±9.8	100–140	0.011
Diastolic blood pressure (mm Hg)	82.1±16.8	70–120	73±8.3	56–80	0.015
ECG (sinus rhythm)	15 (55.5%)		NA		
Atrial Fibrillation	8 (29.6%)		NA		
LV-EF (%)	38.5±18		NA		
LV-FS (%)	22.3±11.7		NA		
Congestive hepatomegaly with ascites	7 (25.9%)		NA		
**Laboratory parameters**					
Serum bilirubin total (mg/dL)	8.5±6.7	2.3 – 30.5	0.73±0.24	0.4–1.2	0
Serum bilirubin direct (mg/dL)	1.56±2.24	0.4 – 11.5	0.18±0.08	0.1 –0.4	0.002
Serum albumin(mg/dL)	2.9±0.39	2 – 3.4	4±0.5	3.1–4.8	0
Serum ALP (IU/L)	176±50.1	113–340	148±48.4	90–260	0.031
Serum ALT (IU/L)	222.7 ±182.38	23–740	20.7±8.7	8 to 38	< 0.001
Serum AST (IU/L)	176±154.4	24–598	19.4±8.3	9–42	< 0.001
Serum LDH (IU/L)	524.8±215.5	210–1071	313±61.2	218–456	< 0.001
Serum Urea (g/L)	86.7±47.8	29–194	31.2±12.9	12–54	< 0.001
Serum creatinin (mg/dL)	1.58±0.74	0.7–3.2	0.65±0.22	0.3– 1	< 0.001
Haemoglobin (g/dL)	10.2±2.3	7–15.1	14.8±1.6	12–17.4	< 0.001
HGF (ng/mL)	**10.09**±**4.84**	2.68 – 21.21	**0.52**±**0.34**	0.033 –1.015	< 0.001

a (IQR, Inter-quartile range; NYHA, New Y°rk Heart Association; JVP, jugular venous pressure; ECG, electrocardiogram; LV-EF, left ventricular ejection fraction; LV-FS, left ventricular fractional shortening).

#### Liver Function Tests

Significant differences were observed in the serum concentrations of liver enzymes, bilirubin (total and direct), urea, creatinine, haemoglobin and HGF between the patient and the control group (*p*<0.05). Hyperbilirubinemia, a feature hepatic dysfunction, was hallmark of almost all patients in this study. Nine patients were presented with total bilirubin level of ≥10 mg/dL with the concentration as high as 30.5 mg/dL and remaining patients had 3–10 mg/dL. Almost all the patients were having hypoalbuminemia, which is a feature of hepatic dysfunction. Based upon liver enzyme patterns in patient sera as well as bilirubin level, the patient group with hepatic abnormality was grouped as either having a hepatocellular profile (n = 19) or a cholestatic profile (n = 8). The amount of HGF in patient sera was significantly higher compared to those of control group (*p*<0.001). Within the patient group, higher concentration of HGF was observed in patients with IHD, which was not significantly different from other patients having VHD, DCM or CHD (*p* = 0.649) (Table S1). However, a strong and significant correlation was observed between HGF level and the total bilirubin level (r = 0.854, *p*<0.001) as assessed by the non-parametric Spearman rank correlation test (Figure S1).

### Hepatic Trans-differentiation of hMSCs in Hepatogenic Conditioned Culture System

#### Cell Proliferation Assay for hMSCs in various Culture Conditions

**Figure 1:**
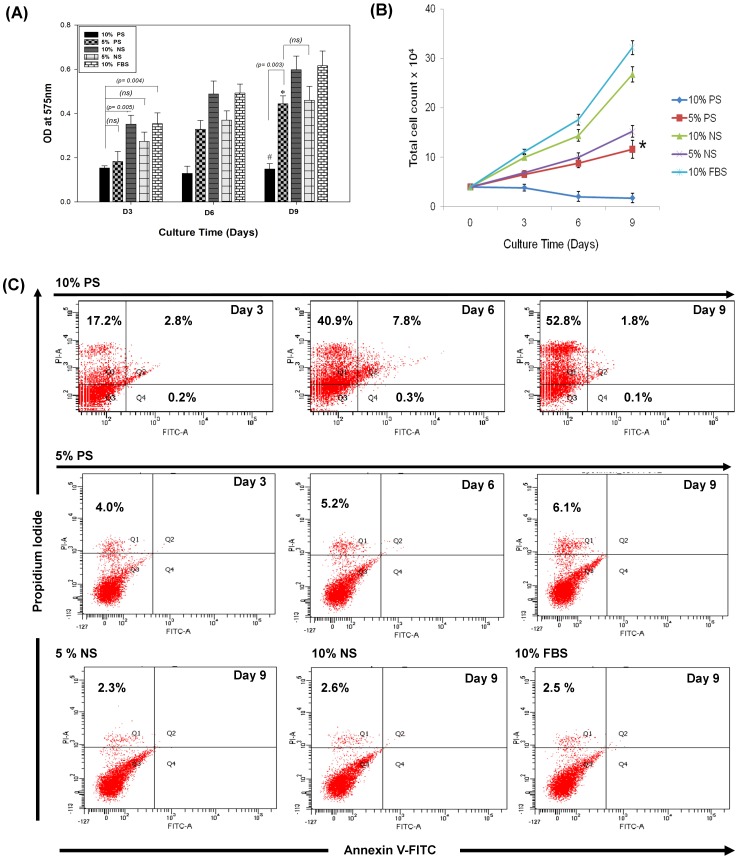
Cell proliferation assay for hMSCs in various hepatogenic culture conditions. (A) *In vitro* cytotoxicity assay showing MTT activity across 9 days of induction in different culture conditions. Error bars represents mean ± S.D. The differences in cell proliferation between the groups were considered statistically significant at *p*<0.05 and *p*-values are indicated on the graph. (*) indicates significant increase in cell proliferation at day 9 compared to day 3 and day 6 in 5% patient sera group. (#) indicates no significant difference in hMSC proliferation at different time points across 9 days of culture. (B) Cell proliferation assay by direct cell counting reveals higher proliferation rate in 10% FBS, which was comparable to that of 10% normal sera (NS) induction group. 10% patient sera (PS) induction group did not show any increase in cell proliferation. However, 5% PS group showed increase in proliferation, which was significantly more compared to 10% PS group (*p<0.05). (C) Cell death assay by annexinV-FITC/PI flow cytometric quantification revealed that 10% patient sera caused mostly necrotic cell death with increase in cell death across 9 days, whereas in 5% PS group cell death was comparably minimal. However, there was negligible cell death in control groups.

MTT assay revealed no significant difference in hMSCs grown in 10% patient sera and 5% patient sera (*p* = 0.505) as well as between 10% patient sera and 5% normal sera (*p* = 0.175) on day 3 (Figure 1A). However, differences were observed in hMSCs proliferation between 10% patient sera and 10% normal sera as well as with 10% FBS on day 3. As the culture time progressed, culture condition using 10% patient sera was found to be cytotoxic, as it did not support further proliferation of hMSCs across 9 days. It was further confirmed by performing direct cell counting under different culture conditions (Figure 1B). Except, 10% patient sera induction group, all other culture conditions, including 5% patient sera induction group, supported significant proliferation (*p*<0.05) over 9 days of culture. No significant difference was observed between cells grown in 5% patient sera induction group and 5% normal sera group. Further, cell death assay was performed by annexin V-FITC/PI flow cytometric assay (Figure 1C), which showed that 10% patient sera caused mostly necrotic cell death revealed by more of propidium iodide (PI)-positive cells and as the day progressed the amount of cell death increased significantly (*p*<0.05). Although there was meagre cell death in 5% patient sera, it was negligible compared to 10% patient sera. From the above findings, we inferred that culture condition containing 10% patient sera was cytotoxic and 5% sera suitably allowed hMSCs proliferation, comparable to that of control sera.

#### Morphological Features of hMSCs-derived Hepatocytes

**Figure 2:**
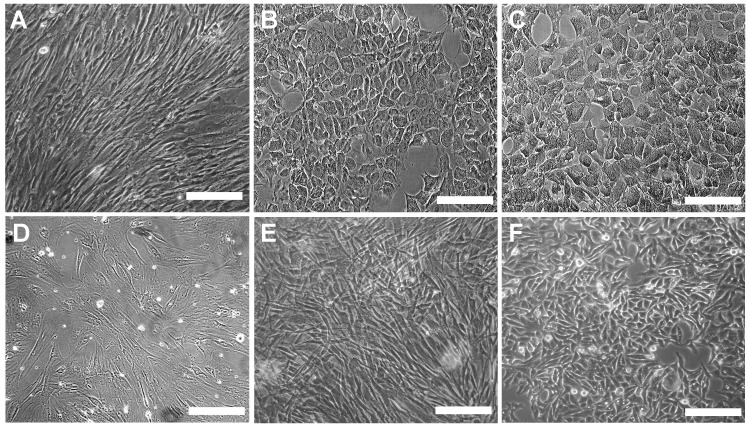
Phase contrast micrographs showing morphological changes observed during hepatic trans-differentiation of hMSCs. (A) hMSCs before hepatic induction, (B) a colony of hMSC derived hepatocytes generated by hepatogenic cocktail induction showing epitheloid morphology after 28 days, (C) polygonal hepatocyte-like colony obtained after induction with patient sera, (D) hMSCs grown in normal sera showing undifferentiated, broad, fibroblastic cells, (E) Untreated hMSCs grown in 10% FBS, showing fibroblastic morphology and (F) an epitheloid colony of HepG2 cells. (Original magnifications × 100) (Scale bar  = 100 μM).

In patient sera induction as well as hepatogenic growth factor cocktail induction group, the process of trans-differentiation was evident by increased nucleo-cytoplasmic ratio, accumulations of cytoplasmic granules and most prominently change in fibroblastic morphology of hMSC to cuboidal, epitheloid morphology of hepatocytes (Figure 2). Islands of compact, round or polygonal-shaped cells (more than 85%) surrounded by spindle-shaped cells with retracting ends and with dense granular cytoplasm were observed during maturation step (Figure 2B and 2C). Approximately, 16±4 hepatocyte-like colonies were formed per 1.5×10^4^ hMSCs in hepatogenic growth factor induction group, whereas 13±5 hepatocyte-like colonies were formed in patient sera induction group after day 28. During the process of trans-differentiation in these two groups, the cell proliferation rate declined as the hMSCs underwent mesenchymal–epithelial transition confirming hepatic specification. However, cells grown in normal control serum as well as in 10% FBS did not show any morphological changes and kept on proliferating (Figure 2D and 2E). HepG2 cells showing epitheloid colonies were used as control (Figure 2F).

#### Real time qPCR Analysis of Hepatocyte Specific Markers in hMSCs- derived Hepatocytes

**Figure 3:**
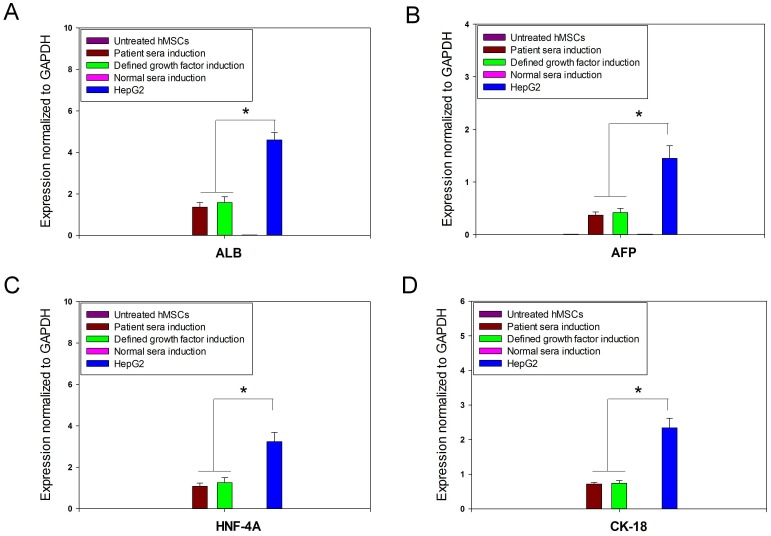
Quantitative real time PCR expression of hepatocyte specific genes. Positive expression of ALB, AFP, HNF-4α and CK18 were observed in patient sera induction as well as hepatogenic cocktail induction group, compared to untreated hMSCs and normal sera induction group. No differences in expressions were observed between the two positive induction groups, but it was significantly lesser compared to HepG2 (*p<0.05). The expressions of genes were normalized to GAPDH and mRNA of untreated hMSCs was used as calibrator.

**Figure 4:**
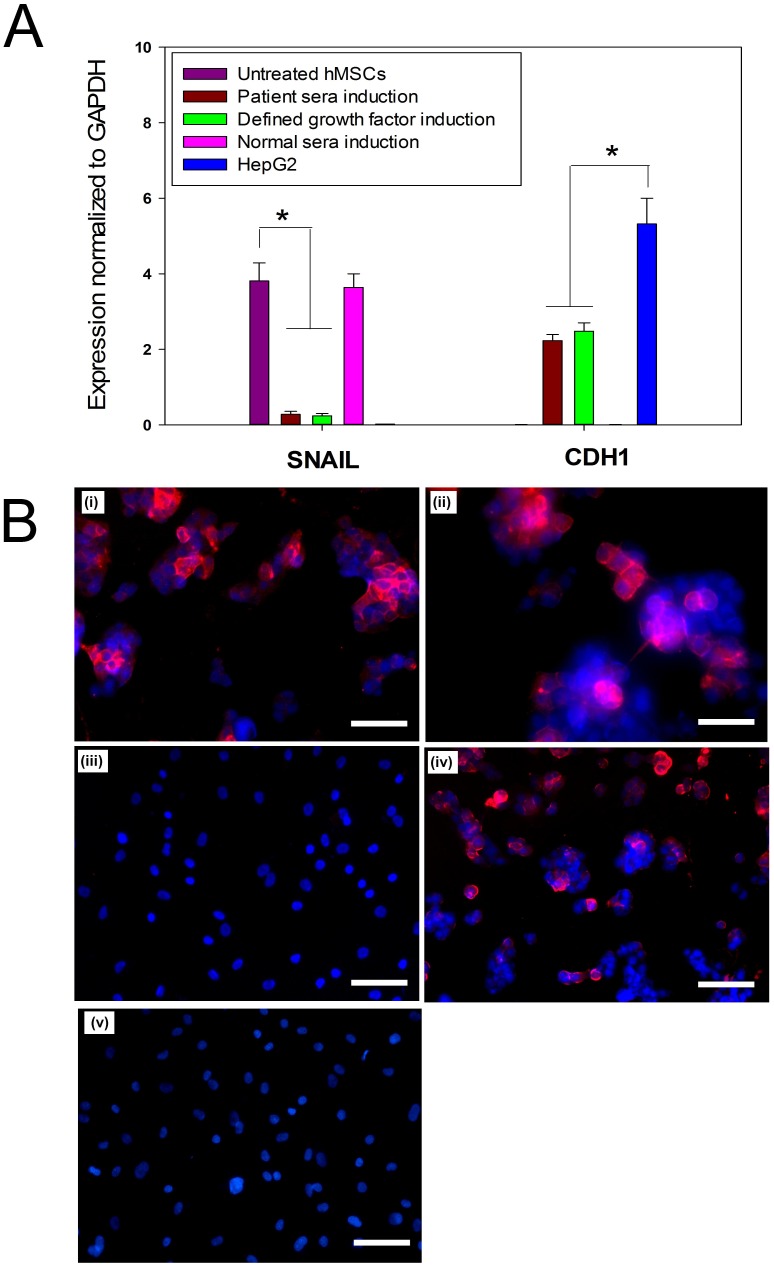
Evaluation of mesenchymal-to-epithelial transition during hepatic trans-differentiation of hMSCs. (A) Real time qPCR analysis for relative quantification of Snail (a mesenchymal marker) and CDH 1 (an epithelial marker) showed that hMSCs-derived hepatocytes in patient sera induction group and growth factor cocktail induction group with a higher expression of CDH1 had low Snail expression. In contrast, cells in normal sera induction group and untreated hMSCs had high expression levels of Snail with very low or no CDH 1 expression. HepG2 cells with high CHD 1 expression had no Snail expression. Statistically significant difference in CDH1 expression was observed between hMSC-derived hepatocytes and HepG2 cells (*p<0.05). Similarly, the difference in Snail expression was statistically significant between hMSCs-derived hepatocytes and untreated hMSCs or normal sera treated hMSCs. (B) Immunofluorescence studies showed positive expression of E-Cadherin in hMSCs derived hepatocytes in growth factor cocktail induction group (i) and patient sera induction group (ii). Normal sera treated hMSCs (iii) and untreated hMSCs (v) were negative for E-Cadherin expression. HepG2 cells were used as positive control (iv). (Original magnifications × 100) (Scale bar  = 100 μM).

Relative quantification of hepatocyte specific genes (AFP, ALB, HNF-4α, and CY-18) by qRT-PCR analysis showed higher expression levels in hMSC-derived hepatocytes in patient sera treatment group as well as defined growth factor treatment group (Figure 3), with no expression observed in untreated hMSCs and normal sera treatment group. Although the difference in expression levels of these hepatic markers was not significant between hMSC-derived hepatocytes in both positive induction groups, it was significantly lower than that of HepG2 cells (*p*<0.05). To confirm the mesenchymal-epithelial transition during hepatic trans-differentiation of hMSCs, we quantified relative expression levels of Snail and CDH 1 mRNA by qRT-PCR. The results revealed significantly higher expression level of Snail in untreated hMSCs and normal sera treated hMSCs compared to hMSCs-derived hepatocytes in patient sera induction group and defined growth factor induction group (*p*<0.05) (Figure 4A). Snail was not detectable in HepG2 cells. In contrast, CDH 1 expression was observed in hMSCs-derived hepatocytes in patient sera induction group and defined growth factor induction group, which was significantly lesser than that of HepG2 cells (*p*<0.05). Untreated hMSCs and normal sera treated hMSCs showed no expression of CDH1.

#### Immunofluorescence and Flow Cytometry Analysis of Hepatic markers

**Figure 5:**
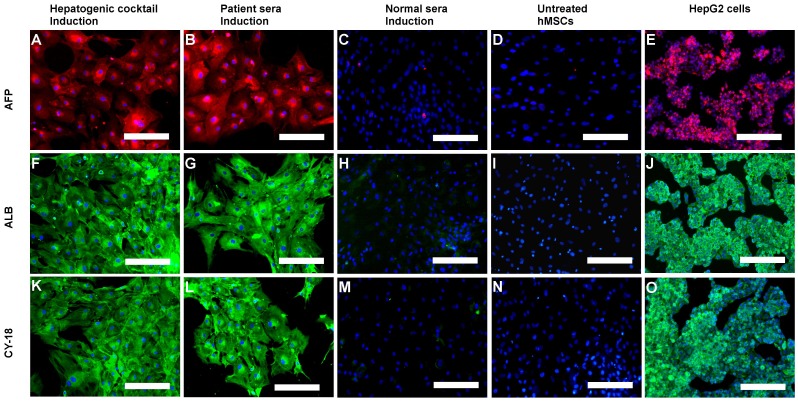
Immunofluorescence analysis of hepatic markers. Immunofluorescence micrographs (merged) showing positive expression of hepatocyte specific markers, AFP (Alexa Fluor 594: red colour), ALB (Alexafluor 488: green colour) and CY-18 (Alexafluor 488: green colour) in hMSCs-derived hepatocyte-like colonies after 28 days of differentiation and negative expression in undifferentiated hMSCs. HepG2 cells were used as positive control. Nuclei were stained with DAPI (blue colour). (Original magnifications × 100) (Scale bars  = 100 μM).

**Figure 6:**
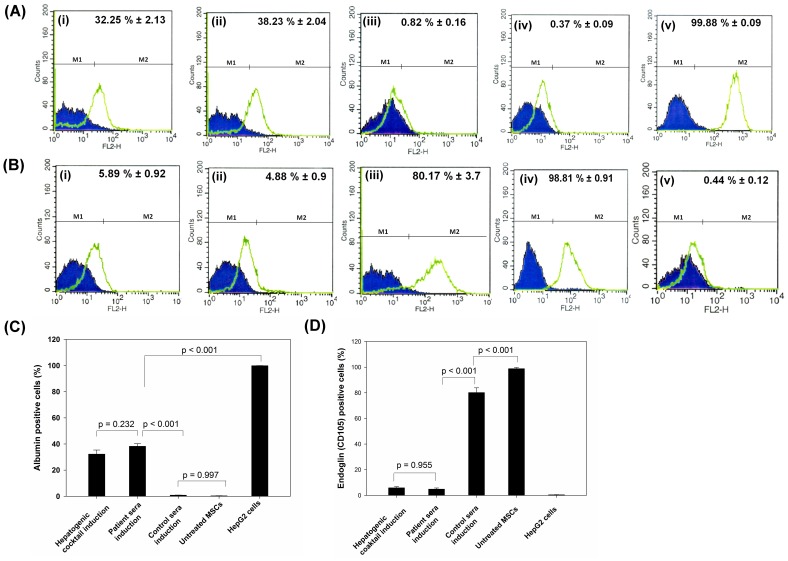
Assessment of hepatic trans-differentiation efficiency of hMSCs. Representative flow cytometry data showing average number of albumin positive cells (A) and endoglin (CD105) positive cells (B) after hepatic trans-differentiation of hMSCs in hepatogenic cocktail induction group (i), patient sera induction group (ii) and normal sera induction group (iii). Untreated hMSCs grown in FBS (iv) and HepG2 cells (v) were used as negative and positive control respectively. Histograms showing isotype control (blue area, quantified by M1) and the positive expression of either albumin or endoglin (green area, quantified by M2). Results are representative of three different experiments. Comparison of the total number of albumin positive cells (C) and endoglin positive cells (D) in different hepatogenic culture conditions as well as controls represents the trans-differentiation efficiency. Differences in cell number between the groups were considered statistically significant at *p*< 0.05 and *p*-values are indicated on the graph.

Strongly positive expression of all three hepatocyte markers (AFP, ALB and CY-18) was observed in hMSCs-derived hepatocytes in patient sera induction group and hepatogenic cocktail induction group, which was comparable to that of HepG2 cells (Figure 5). However, expression of these hepatocytes-specific markers in untreated hMSCs as well as those grown in normal sera was negligible. Expression of E-Cadherin was observed in hMSCs derived-hepatocytes in both patient sera induction group and growth factor cocktail induction group (Figure 4B), but not in normal sera induction group and untreated hMSCs. Flow cytometric analysis showed that 32±2.13% cells were albumin positive in hepatogenic cocktail group (Figure 6Ai) and 38.23±2.05% (Figure 6Aii) cells in patient sera induction group and the difference between them was insignificant (Figure 6C). The hMSCs grown in normal sera and untreated hMSCs were negligibly albumin-positive, accounting for 0.82% and 0.37% of total cells respectively (Figure 6Aiii and 6Aiv). Since, hepatic transdifferentiation efficiency was only 30–35%, expression of endoglin (CD105), a cell surface marker of undifferentiated mesenchymal stem cell, was further assessed. In hepatocytes observed, Approximately, 5.89% and 4.88% endoglin-positive cells were observed in hepatogenic cocktail induction group and patient sera induction group respectively (Figure 6Bi and Bii), and difference between them was statistically insignificant (Figure 6D). HepG2 cells, an endoglin-negative human hepatoma cell line [38], showed only 0.44% endoglin expression (Figure 6Bv), which was significantly different from that of hepatocytes in patient sera induction group (*p* = 0.077), but not from hepatogenic cocktail induction group (*p* = 0.026). However, hMSCs grown in normal sera as well as untreated hMSCs showed 80.17% and 98.81% endoglin-positive cells, after 28 days of culture (Figure 6Biii and 6Biv).

#### Liver Function Tests for hMSC-derived Hepatocytes

**Figure 7:**
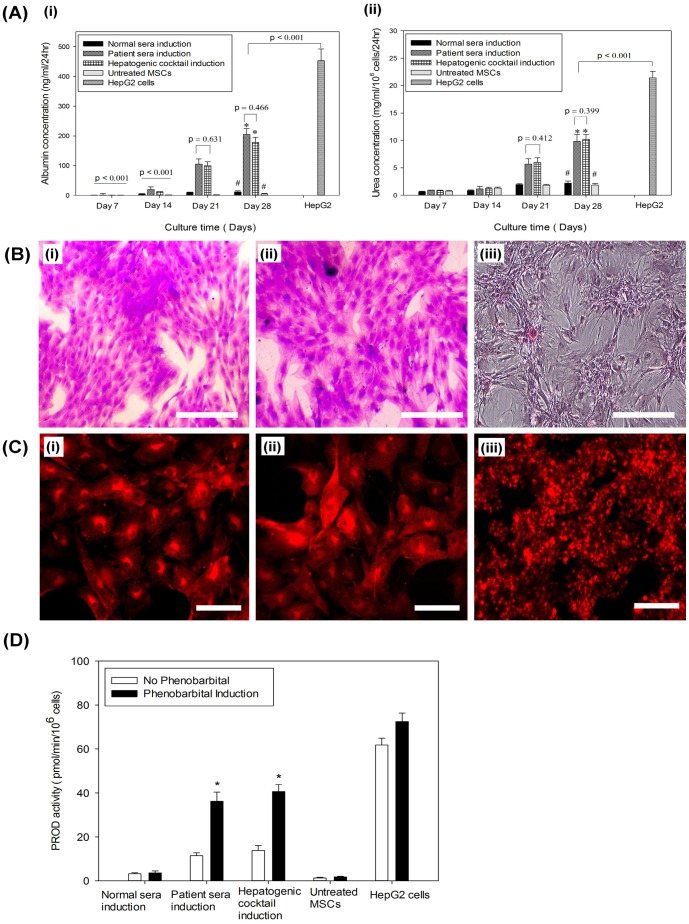
Functional characterization of hMSCs-derived hepatocytes. (Ai) Albumin ELISA of the culture media collected from various hepatic induction groups and controls. Differences in albumin concentration between the groups were considered statistically significant at *p*<0.05 and *p*-values are indicated on the graph. (*) indicates significant increase in albumin release by hepatocytes in patient sera induction group as well as cocktail induction group at day 28 compared to their day 21 counterparts. (#) represents no significant difference in albumin concentration in undifferentiated hMSCs of normal sera induction group and untreated hMSCs at 28 compared to earlier days. (Aii) Urea assay showed higher concentration in hMSCs derived hepatocytes compared to controls on day 28, but was less than that of HepG2 cells. (B) Glycogen accumulation was represented by PAS staining, which showed positive expression (magenta color) in hMSCs-derived hepatocyte colonies of patient sera induction group (i) and defined growth factor induction group (ii). Normal sera treated hMSCs were PAS negative (iii). (Original magnifications, ×100) (Scale bars  = 100 μM). (C) PROD Assay for phenobarbital inducible cytochrome P450 was confirmed by positive expression (red fluorescence) in hMSC-derived hepatocytes in patient sera induction group (i) and growth factor cocktail induction group (ii). HepG2 cells were used as positive control (iii). (Original magnifications × 100) (Scale bars  = 100 μM). (D) Spectrofluorimetric quantification of PROD activity revealed a significant increase in fluorescence intensity after phenobarbital induction in hMSC-derived hepatocytes in patient sera induction group and defined growth factor induction group (* *p*<0.05). However, negligible PROD activity was observed in untreated hMSCs and normal sera treated hMSCs even after phenobarbital induction. HepG2 cells with higher level of fluorescence activity compared to hMSC-derived hepatocytes did not show significant elevation in PROD activity after phenobarbital induction.

Albumin release was negligible during first two weeks, which increased significantly on day 21 in patient sera induction group and growth factor induction group compared to normal sera induction group and untreated MSCs (*p*<0.05) (Figure 7Ai). Further increase in albumin release was observed on day 28 compared to day 21 in both patient sera induction group and growth factor induction group, although difference between them was insignificant (*p* = 0.466). Urea assay also reveals that hMSCs derived hepatocytes in both patient sera induction group and growth factor cocktail induction group had higher concentration on day 28 compared to controls, but no significant difference was observed between them (*p*< 0.05) (Figure 7Aii). Similarly, accumulation of intracellular glycogen was evident by positive PAS staining in hepatocyte colonies obtained in patient sera induction group and growth factor treatment group after day 28, whereas normal sera treated hMSCs were PAS negative (Figure 7B). The number of PAS-positive cells in patient sera induction group was 36±7% compared to 31±3% in hepatogenic growth factor induction group, exhibiting almost similar pattern of maturation. The liver function tests of hMSCs-derived hepatocytes were consistent with the morphological changes and gene expression study. To assess whether the hMSCs-derived hepatocytes in patient sera induction group and growth factor induction group possess inducible cytochrome P450 variant for drug metabolism, PROD assay was carried out before and after phenobarbital induction. Higher number of PROD positive hepatocytes were observed in patient sera induction group and growth factor induction group under fluorescent microscope (Figure 7C), whereas very low or no PROD activity was observed in untreated hMSCs and normal sera induction group (data not shown). Spectrofluorimetric quantification of PROD activity showed a significant increase in fluorescence intensity after phenobarbital induction in hMSCs-derived hepatocytes in patient sera induction group and growth factor cocktail induction group (*p*<0.05) (Figure 7D), revealing presence of inducible cytochrome P450 system. Although HepG2 cells had higher level of resorufin fluorescence compared to hMSCs-derived hepatocytes, it was not elevated significantly after phenobarbital induction, may be due to drug insensitivity. No increase in PROD activity was observed in untreated hMSCs and normal sera treated hMSCs even after phenobarbital induction.

## Discussion

In this study, severe liver function abnormalities were observed in a well-defined cohort of patients with various cardiac diseases leading to chronic heart failure. The LFTs are mostly of cholestatic pattern (70.3% of patients) with severe increase in bilirubin and ALP levels (Table 1), which corroborates with earlier findings [4], [39]. Remaining patients showed hepatocellular LFTs represented by elevated serum ALT and AST levels and hyperbilirubinemia. Such cardiac hyperbilirubinemia has been associated with poor outcome in chronic cardiac failures [40], [41], which may arise due to hepatocellular damage from decreased perfusion, cholestatic liver damage from increased central venous pressure (CVP), sluggish flow of bile, inspissated bile and bile duct calculi and low left ventricular ejection fraction (LV-EF) leading to venous congestion [4], [5], [42]. Interestingly, high level of bilirubin is associated with a decreased risk of coronary heart disease (CHD) and cardiovascular disease (CVD) [43]. While the full spectrum by which bilirubin acts to protect against CVD is not fully understood, earlier reports suggests that bilirubin protects against oxidative stress by acting as an anti-oxidant and free radical scavenger with anti-atherogenic properties [44]. In this study, the increased level of this component serves as a potent prognostic biomarker for complex cardiovascular diseases like IHD, VHD, CHD and DCM. Sera obtained from these patients also exhibited increased level of HGF, as reported in many other liver diseases such as hepatitis, cirrhosis and hepatic failure [29], [30], [45]. HGF promotes liver regeneration and prevent hepatic fibrosis caused due to tissue damage [46]. Moreover, a strong correlation was observed between the total bilirubin and the HGF levels in the present study, which signifies that elevation in serum HGF level was directly related to the severity of cardiovascular disease. Besides the liver, serum HGF elevation occurs during the early phases of both acute myocardial infarction (AMI) and heart failure (HF) [31], [47], [48]. Therefore, it is important to determine the sources of HGF in this study, as both the heart and liver showed abnormal function and would have released HGF upon damage. It is known that enhanced level of HGF in serum may be either due to low hepatic clearance or elevated synthesis of HGF [49]. The elevated serum HGF level in this study may be caused by either the attempt to regenerate new hepatocytes in damaged liver, or the decrease rate of clearance by liver, which require further studies for confirmation. Since there is marked increase in serum HGF level in this study, we hypothesize that HGF may play a key role as a prognostic biomarker of cardiac failure-associated jaundice (hyperbilirubinemia).

Cardiac-failure-associated jaundiced sera containing higher level of HGF was optimized as a hepatogenic conditioned culture system for hMSCs trans-differentiation. Our initial findings demonstrate that 10% sera were cytotoxic, while 5% sera suitably supported proliferation as well as hepatic trans-differentiation of hMSCs, which corroborates earlier reports [24], [26]–[28], [50]. It has also been reported that conditional selective medium containing cholestatic serum provides selective proliferative signals for hepatocytes, simultaneously eliminating non-hepatocyte-like cells [26], [50]. Interestingly, HGF is known to have potential anti-necrotic and anti-apoptotic effect on hepatocytes in cholestatic jaundice and rescues them from damage [51]. Since the cardiac jaundiced sera possess higher concentration of HGF, we assume that 5% sera induced hepatic differentiation of hMSCs with no significant effect on cell viability. Although, 10% sera also contain higher concentration of HGF, its anti-apoptotic and anti-necrotic effects would have been suppressed by the higher concentration of bilirubin and bile acids [52], [53]. Nevertheless, high serum level of HGF released from damaged heart/liver in such cases could effectively potentiate hepatic induction, comparable to that of known recombinant hepatogenic factors [20]–[22].

Quantitative gene expression studies as well as immunofluorescence analysis revealed that hMSCs-derived hepatocytes expressed mature markers like albumin and cytokeratin-18 by 28 days, which were not detected by day 14. The hMSCs treated with jaundiced sera generated approximately 38% albumin-positive cells, which was comparable to 32% in case of sequential induction with known hepatogenic growth factors and cytokines. Our data were almost consistent with earlier reports of hepatic trans-differentiation of commercially available hMSCs [54]. However, we could not achieve albumin positive cells as high as ≥ 81% in case of ESCs/iPSCs-derived hepatocytes [55]. This decrease in hepatic trans-differentiation efficiency of hMSCs may be due to mesenchymal-to-epithelial transition followed by hMSCs compared to highly efficient hepatic differentiation of ESCs/iPSCs mimicking normal liver development process [56]. This phenomenon of mesenchymal-to-epithelial transition was confirmed by decreased expression of Snail, a mesenchymal marker and increased expression of CDH1, an epithelial marker in the hMSCs-derived hepatocytes in patient sera and cocktail induction groups. Since the trans-differentiation efficiency was below 40%, the obvious question arises regarding the fate of albumin-negative cells as to whether they still possess the hMSC phenotype. In this regard, endoglin (CD105)-positive fractions were quantified in hepatocyte-like colonies, which represent as low as around 5% populations in both patient sera induction as well as cytokine induction group. This implies that hMSC phenotype was almost lost during hepatic differentiation and these alb^−^/endoglin^−^ cells might be the precursors of hepatocytes, which requires further characterization. The hMSCs-derived hepatocytes using patient sera in this study showed *in vitro* functional features characteristics of liver cells such as albumin synthesis, urea release, glycogen storage with phenobarbital-inducible cytochrome P450 activity. One major advantage of using patient sera over commercially available growth factors might be clinical transplantation of hMSCs-derived hepatocytes. It is because the commercially available growth factors are of bacterial origin and presence of endotoxin (as lipopolysaccharide) might make their clinical usage unsuitable. It is reported that endotoxins present in commercially growth factors (as low as 0.0005%) can have a negative impact on differentiation potential of stem cells [57]. Patient sera contain natural source of endotoxin free-HGF, which can be used for differentiating hMSCs towards generation of autologous hepatocytes and can be applied clinically in future.

In conclusion, our findings report that hMSCs can effectively trans-differentiate towards functional hepatocytes *in vitro* using a novel hepatogenic conditioned culture system containing sera from cardiac-failure-associated congestive/ischemic liver patients. These hMSCs-derived hepatocytes might be clinically relevant for autologous cellular therapy during end-stage liver failure as well as for *in vitro* toxicity testing.

## Supporting Information

Figure S1 **Correlation between serum HGF levels and total bilirubin level in cardiac failure-associated jaundiced patients and volunteer donors.**
(TIF)

Table S1 **Liver function abnormality and serum HGF levels in heart failure patients.**
(DOCX)

Table S2 **The genes and primer sequences used in real-time qPCR.**
(DOCX)
