# Supplementary material for: In Vitro Hepatic Trans-Differentiation of Human Mesenchymal Stem Cells Using Sera from Congestive/Ischemic Liver during Cardiac Failure
Source: PLoS One. 2014 Mar 18;9(3):e92397. doi: 10.1371/journal.pone.0092397 (PMC3958528; doi:10.1371/journal.pone.0092397)
Supplement: Table S1 — Liver function abnormality and serum HGF levels in heart failure patients. (DOCX) [file pone.0092397.s002.docx]

**Table S1. Liver function abnormality and serum HGF levels in heart failure patients.**

| **Heart failure type (n)** | **Liver function abnormalities** | | **HGF level (ng/mL) *^a^*** |
| --- | --- | --- | --- |
|  | **Hepatocellular profile (n)** | **Cholestatic profile**  **(n)** |  |
| Ischemic heart disease (8) | 3 | 5 | 12.07 ± 5.2 |
| Valvular heart disease (11) | 3 | 8 | 9.18 ± 1.5 |
| Dilated cardiomyopathy (3) | 0 | 3 | 11.05 ± 5.03 |
| Congenital heart disease (5) | 2 | 3 | 9.6 ± 1.3 |

*^a^* *p* value = 0.649 in between HGF levels of various heart failure groups
