# Supplementary material for: In Vitro Hepatic Trans-Differentiation of Human Mesenchymal Stem Cells Using Sera from Congestive/Ischemic Liver during Cardiac Failure
Source: PLoS One. 2014 Mar 18;9(3):e92397. doi: 10.1371/journal.pone.0092397 (PMC3958528; doi:10.1371/journal.pone.0092397)
Supplement: Table S2 — The genes and primer sequences used in real-time qPCR. (DOCX) [file pone.0092397.s003.docx]

**Table S2. The genes and primer sequences used in real-time qPCR.**

| Gene Name*^a^* | Primer sequence (5´- 3´) | Product size (bp) | NCBI accession number |
| --- | --- | --- | --- |
| AFP | F: AAATGCGTTTCTCGTTGC  R: GCCACACGGCCAATAGTTTGT | 136 | NM_001134.1 |
| HNF-4α | F: ATGACAATGAGTATGCCTACCT  R: GGTCGTTGATGTAGTCCTCC | 131 | NM_178850.1 |
| ALB | F: CGCTATTAGTTCGTTACACCA  R: TTTACAACATTTGCTGCCCA | 101 | NM_000477.5 |
| CY-18 | F: CCCGTCACGCCCTACAGAT  R: ACCACTTTGCCATCCACTATCC | 171 | NM_000224.2 |
| CDH 1 | F: TTAAACTCCTGGCCTCAAGCAATC  R: TCCTATCTTGGGCAAAGCAACTG | 139 | XM_005256272.1 |
| SNAIL | F: CGCGCTCTTTCCTCGTCAG  R: TCCCAGATGAGCATTGGCAG | 181 | NM_005985.3 |
| GAPDH | F: GCACCGTCAAGGCTGAGAAC  R: GGATCTCGCTCCTGGAAGATG | 73 | NM_002046.3 |

*^a^* F: forward primer; R: reverse primer; ALB: albumin; AFP: α-fetoprotein; HNF-4α: hepatocyte nuclear factor-4α; CY-18: cytokeratin 18; CDH 1: E-Cadherin; GAPDH: glyceraldehydes-3-phosphate dehydrogenase
